# Estimation of the Band Gap of Carbon Nanotube Bundles

**DOI:** 10.3390/ma17071530

**Published:** 2024-03-27

**Authors:** Yi Ding, Jing-Zhe Chen

**Affiliations:** School of Science, Shanghai University, Shanghai 200444, China

**Keywords:** single-walled carbon nanotubes, carbon nanotube bundles, first principles, band gap, deformation

## Abstract

The electronic structure of carbon nanotube bundles (CNTBs) can be a tough task for the routine first-principle calculation. The difficulty comes from several issues including the atomic structure, the boundary condition, and above all the very large number of atoms that makes the calculation quite cumbersome. In this work, we estimated the band gap of the CNTBs based on the results from single-walled carbon nanotubes (SWCNTs) under different deformations. The effects of squeezing, stretching, and torsion on the bands of SWCNTs were investigated through first-principle calculations, from which the band gaps of bundles were analyzed because the effects of these deformations were qualitatively independent when the distortions were small. Specifically, the gaps of (4,4) and (8,0) CNTBs under a reasonable torsional strength were predicted, wherein we were able to see metal–semiconductor and semiconductor–metal transitions, respectively. Such reversible mechanical modification of the conductivity may be helpful to the future band-gap engineering in nanoscale circuits.

## 1. Introduction

Since the discovery of carbon nanotubes (CNTs) [1,2], their excellent physical properties including strong structural stability and super high electron mobility have attracted a lot of attention of researchers [3,4,5,6,7,8]. Therefore, CNTs are very good candidates of the next generation of connecting wires for nanoscale circuits. For multi-walled carbon nanotubes (MWCNTs), their conductivity is mostly dominated by the outmost layer [9,10], meaning that SWCNTs turn out to be the irreducible fundamental components [11] in the circuits. The conductivity of SWCNTs at equilibrium is determined by one pair of parameters (n,m) of the structural configuration, namely, the chiral index. Recently, CNTBs have attracted researchers’ interests, and some efforts have been made regarding the mechanical resilience [12,13] and fracture patterns [14] under torsional strain. Singh [15] used CNTBs to build hybrid CMOS (complementary metal-oxide semiconductor)-compatible devices, among which the conductivity of CNTBs is one of the key issues to study. However, the routine first-principle methods such as density functional theory (DFT) or Hartree–Fock are not capable of handling a complex system like this from the atomic scale. Here, we treat the CNTBs as a whole group consisting of several deformed SWCNTs by analyzing each of them under particular deformation, wherein we were able to estimate the band gap change of the bundle during the deformation.

There are many feasible ways to tune the energy gap of carbon tubes. Some of them are chemical solutions such as element doping [16], vacancies doping [17,18,19], or atomic absorption [20,21]. As far as physical solutions are concerned, applying an external field has been reported to achieve this goal as well [22]. What is more, the mechanical way is also a possible choice. Due to their structural robustness, SWCNTs can withstand a relatively large degree of deformation before breaking down; hence, different types of mechanical deformation can also be utilized for band gap engineering. Some previous works verified that SWCNTs’ conductivity can be changed to a relatively large extent when they are radically compressed [23,24,25,26,27,28] or axially torqued [29,30], and sometimes a semiconductor–metal transition can even be observed.

In this paper, using density functional theory (DFT), we first investigated three types of deformations, namely, flattening, tension, and torsion, within a reasonable degree on six armchair or zigzag SWCNT samples. These different deformations showed different effects on the band gap tuning, depending on the tube chirality, which can be concluded as the following: metal SWCNTs are sensitive to torsion, which tends to open the gap, while they are reluctant to flattening and tension, and semiconducting SWCNTs’ gap can be closed by flattening, being sensitive to tension and torsion, but the trend depends on the particular chirality. When the distortion is small, the effects from different deformations are roughly independent of each other [31]; combining them together, we estimate the band gap of (4,4) and (8,0) CNTBs, and significant change can be seen from the straight bundle to the torsional.

## 2. Model and Methodology

**Figure 1:**
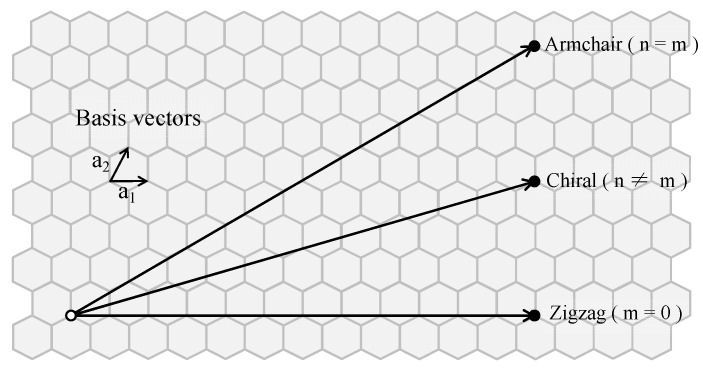
SWCNTs (n,m): Roll up by connecting two carbon atoms (○,●).

SWCNTs can be constructed by curling flat graphene and are usually represented by (n,m), which determines the SWCNT characteristics. As shown in Figure 1, the chiral indices (n,m) define the chiral vector (black arrows) that connects two carbon atoms in a graphene plane, and they are represented as na**_1_** + ma_2_, where a_1_ and a_2_ are the basis vectors. The electronic structure of the deformed SWCNTs are evaluated with the density functional theory (DFT) method. The systems are optimized before the final band calculation, and we assume that external contact, which sometimes is necessary for the deformation, can be neglected when evaluating the bands. The details of the calculation setting are introduced as the following:

Projector augmented wave (PAW) pseudopotential is adopted to handle the effect of inner core electrons [32], and the Perdew–Burke–Ernzerhof (PBE) [33] functional is chosen to calculate the exchange-correlation energy. Plane wave basis has an energy cutoff of 300 eV; the k-point sampling (1 × 1 × k_Z_) is listed in Table 1, where the k-point is the quantum number of an electronic system moving in a crystal lattice, being a vector on the reciprocal space, and it is the sampling point in the concrete calculation. In plane of the cross-section, there is a large vacuum distance between two adjacent tubes; hence, they do not interact with each other, and setting the corresponding k-point sampling 1 is sufficient. Along the longitudinal axis, the k-point is set to a large integer k_Z_, and the exact number depends on the cell size. The electron density is converged with the criteria that the difference of total energy between two adjacent iterative steps is less than 10^−4^ eV. In structural optimization, conjugate gradient approximation [34] is used for the search of the energy minimum, where the iterations stop when the maximum force on each atom is less than 0.05 eV/Å. The model was constructed using Materials Studio [35], and the structure was optimized with DS-PAW in the Device Studio program [36].

## 3. Results and Discussion

In this section, we first check the effects of three different deformations on the band gap for SWCNTs. They are summarized separately in the following for simplicity.

### 3.1. Flattened Case

**Figure 2:**
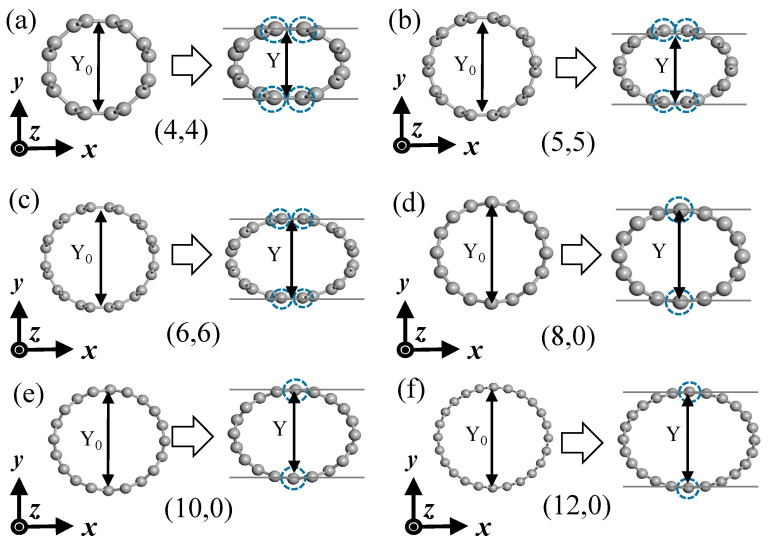
SWCNT structure schematics in the cases of flattening. (**a**) (4,4) SWCNT; (**b**) (5,5) SWCNT; (**c**) (6,6) SWCNT; (**d**) (8,0) SWCNT; (**e**) (10,0) SWCNT; (**f**) (12,0) SWCNT.

We used a dimensionless flatness parameter ε_yy_ − $\frac{Y_{0}-Y}{Y_{0}}$ to quantify the flattening strength of SWCNTs, where Y_0_ is the diameter of the original tube and Y is the diameter of the deformed tube in the Y-axis direction, as shown in Figure 2. We moved the carbon atoms in the dashed circles in the y directions and fixed them; then, we relaxed the remaining part to obtain the final structure of the flattened case. The parameter ε_yy_ varied from 0.0 to 0.4 with intervals of 0.1 during this process. The corresponding external forces were considered practically reasonable according to the work by Mazzoni [23]; the force per unit length of the nanotube that was necessary to reach the insulator–metal transition was 7.4 N/m.

**Figure 3:**
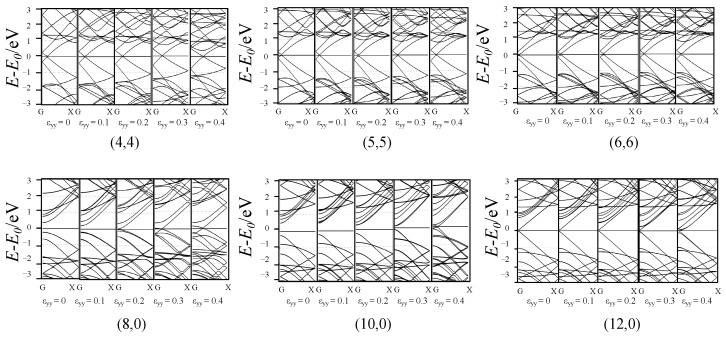
Band structure of the SWCNTs after flattening.

Figure 3 shows the change in the band structure of SWCNTs after flattening, where G and X represent two highly symmetric points in the Brillouin region of the carbon nanotube with the coordinates [0,0,0] and [0.5,0,0], respectively. The horizontal axis represents the k vector, and the vertical coordinate represents the difference between the electron energy and the Fermi level. During the deformation of flattening, for armchair SWCNTs, the crossing point of the bands near the Fermi level moved a little towards “X” point, and the effect of hybridization was stronger. For zigzag SWCNTs, the band gap of the semiconducting tubes tended to close during the deformation, and it was even able to be closed completely when ε_yy_ reached 0.2. The situation of the metallic tube did not change very much with a closed gap when ε_yy_ reached 0.4.

**Figure 4:**
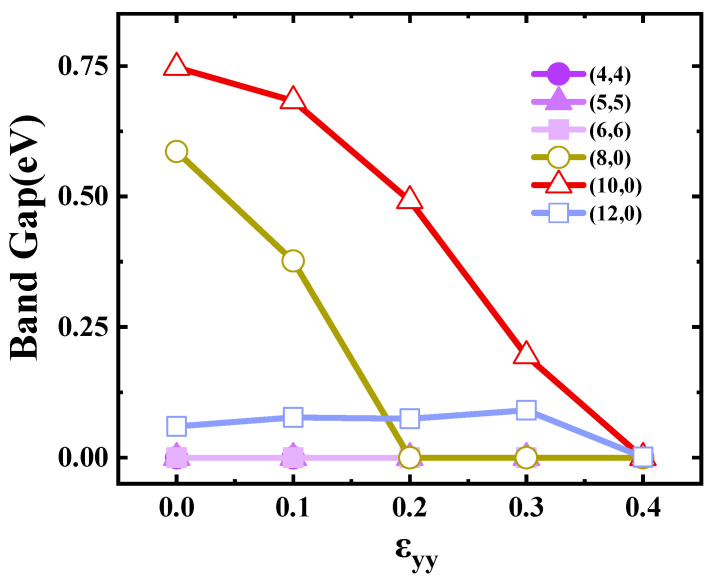
Band gaps of the SWCNTs as a function of ε_yy_.

**Figure 5:**
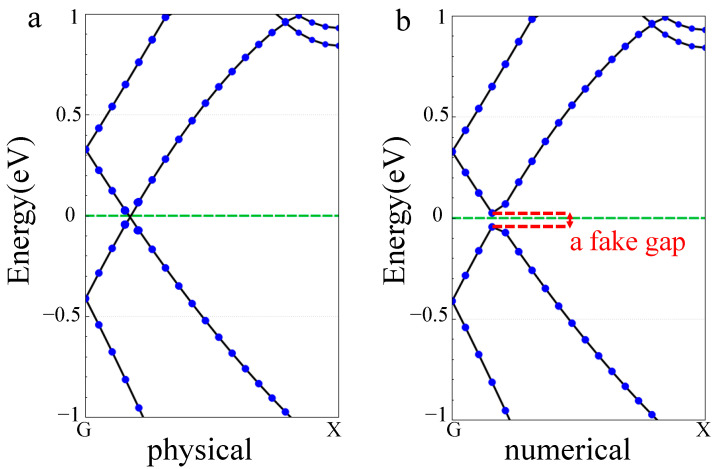
Band structure of (4,4) SWCNTs. (**a**) Physical; (**b**) numerical.

Figure 4 shows the effect of flattening on the SWCNTs’ band gap. We can see that the band gap of armchair SWCNTs had no significant change. There are some works [37,38] reporting that the armchair SWCNT has a finite band gap during the flattening process, whereas from the system symmetry, we can deduce that the small gap was not physical but numerical, as illustrated in Figure 5. This was the band structure of (4,4) SWCNTs at a flattening strength of 0.1. When the number of calculated K points is finite, a fake gap can be drawn by plotting software at the crossing point of the bands. The band gaps of (8,0) and (10,0) tubes showed a monotonic decrease when the flattening strength was increased, wherein the tubes changed from semiconductor-type carbon tubes to metal-type carbon tubes. The band gaps of (12,0) SWCNTs increased slightly and eventually decreased to 0 eV. In this process, the carbon tube was always metallic.

**Figure 6:**
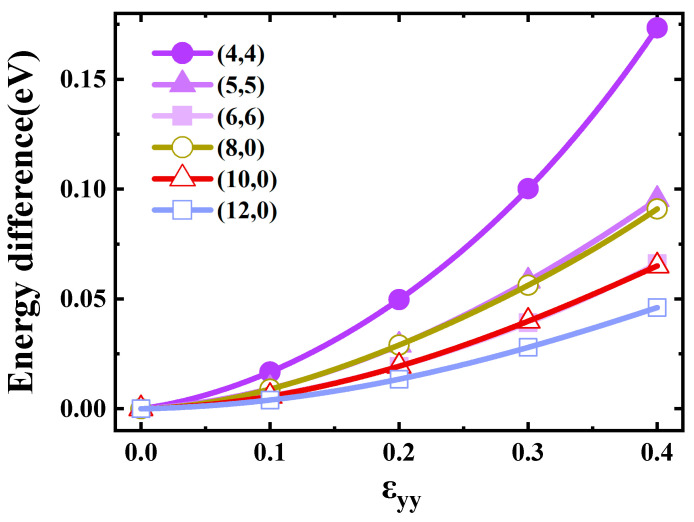
Deformation energy of the SWCNTs as a function of ε_yy_.

Figure 6 displays the fitting diagrams for the energy change of carbon atoms post-deformation. The deformation energy was obtained by counting the difference between the total energy of the different optimized systems and dividing the energy change by the number of atoms to obtain the average energy change per carbon atom. The energy curves were fitted with quartic polynomials. The choice of polynomial degrees in the fitting process is primarily dictated by the pursuit of accuracy, as established in the work of Kinoshita et al. [39]. With the increase in the flattening strength, the energy of all carbon atoms increased, among which the energy change of (4,4) was the most obvious. When the extrusion strength was 0.4, the energy of a carbon atom increased by 0.17 eV, and the increase of (12,0) was the least, wherein the energy of a carbon atom increased by 0.05 eV. If the diameter of the carbon tube is larger, the relative displacement of the carbon atoms will be smaller, that is, the energy change will be smaller.

**Figure 7:**
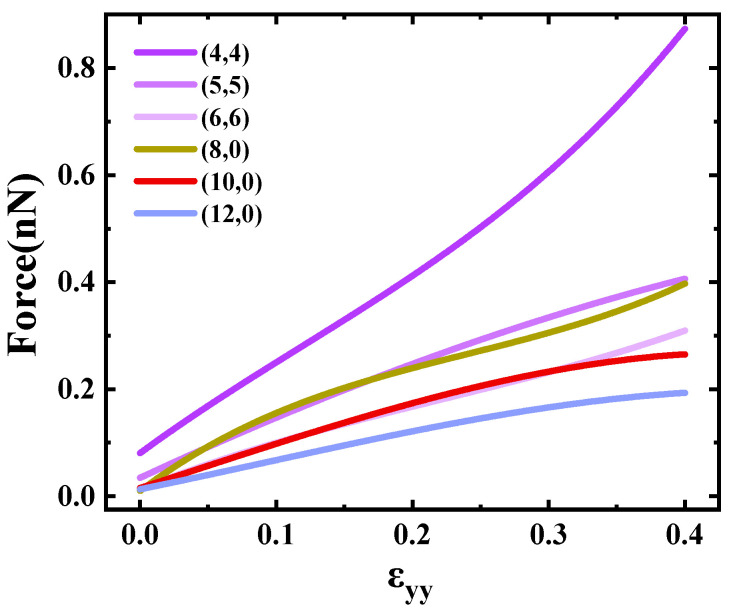
Forces required to flatten SWCNTs.

The force acting on the atom can be derived as the first-order derivative of the flattening curves [39]. The relationship between deformation strength and atomic force is presented in Figure 7. Observing Figure 7, it is evident that the force increased with the augmentation of deformation strength, and the force necessitated for flattening was comparatively smaller than that required for tension and torsion. It is noteworthy that for identical types of SWCNTs, the deformation force exhibited an increase with a decrease in diameter when subjected to the same deformation strength. For the (4,4) carbon tube, the force required for extrusion was the most: when the flattening strength was 0.4, the force required was 0.9 nN; for (12,0), the force required was the least: when the flattening strength was 0.4, the force required was 0.2 nN.

### 3.2. Tensioned Case

**Figure 8:**
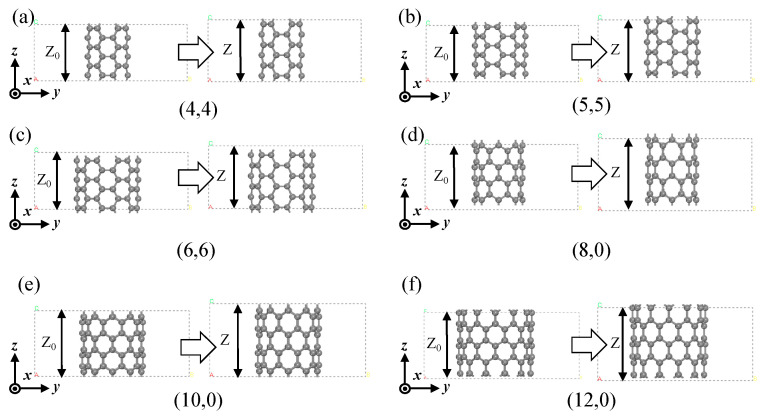
SWCNT structure schematics in the cases of tension. (**a**) (4,4) SWCNT; (**b**) (5,5) SWCNT; (**c**) (6,6) SWCNT; (**d**) (8,0) SWCNT; (**e**) (10,0) SWCNT; (**f**) (12,0) SWCNT.

We used a dimensionless tensile degree ε_zz_ − $\frac{Z-Z_{0}}{Z_{0}}$ to quantify the tensile strength of SWCNTs, where Z_0_ is the length of the original tube and Z is the length of the deformed tube in the Z-axis direction, as shown in Figure 8. The diagram shows six SWCNTs from the intrinsic carbon tube to a tensile strength of 0.1. In this paper, the parameter ε_zz_ varied from 0.0 to 0.1 with intervals of 0.025 during this process.

**Figure 9:**
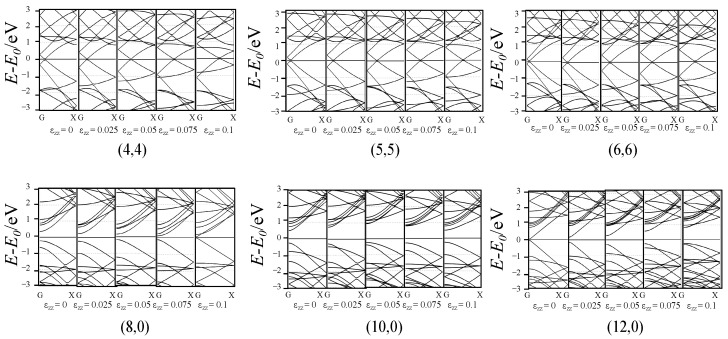
Band structure of the SWCNTs after tension.

Figure 9 shows the tension effect on the band structure. For armchair SWCNTs, the crossing point of the bands near the Fermi level moved towards “X” point and the tube remained metallic. For zigzag SWCNTs, the band gap of the tube tended to close for semiconducting tubes and open for metallic ones during the deformation.

**Figure 10:**
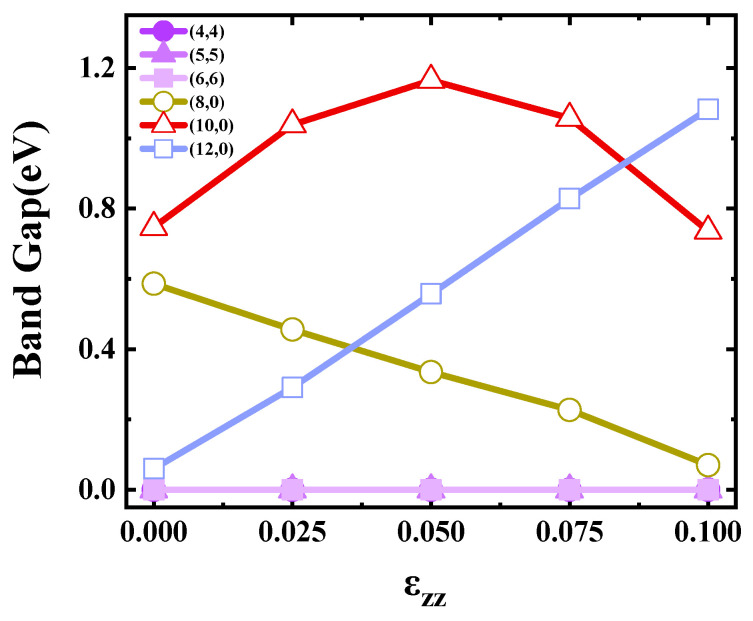
Band gaps of the SWCNTs as a function of ε_zz_.

Figure 10 shows the effect of tension on the SWCNTs’ band gaps. The band gaps of armchair SWCNTs underwent no variation, while distinct band-gap behaviors were evident among the three types of zigzag SWCNTs. Specifically, the band gap of the (8,0) tube showed a monotonic decrease (0.6 eV→0.07 eV) with increasing tensile strength, wherein the (8,0) tube changed from a semiconductor-type carbon tube to a metal-type carbon tube. The (12,0) SWCNTs showed a monotonic increase (0.06 eV→1.0 eV) with increasing tensile strength, wherein the (12,0) tube changed from a metal-type carbon tube to a semiconductor-type carbon tube. For the (10,0) tube, the band gap’s trend tended to be a symmetric parabola with the peak at approximately (ε_zz_ = 0.05). The (10,0) tube was always a semiconductor carbon tube with increasing tensile strength, but the band gap reached its maximum value at (ε_zz_ = 0.05). The change of the band gap of the stretched carbon tube can be explained by the theory of Yang [31].

**Figure 11:**
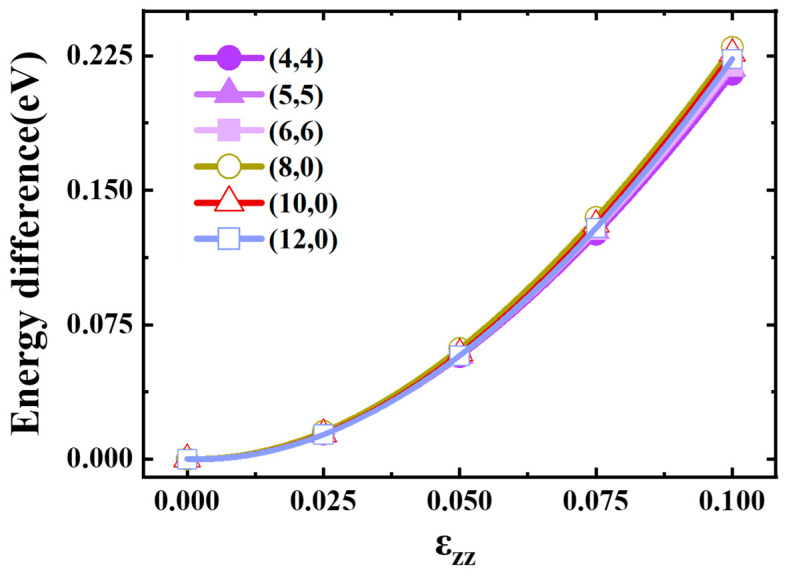
Deformation energy of the SWCNTs as a function of ε_zz_.

Figure 11 displays the fitting diagrams for the energy change of carbon atoms post-deformation. It can be seen from the figure that for all kinds of carbon tubes, the energy change after stretching was independent of the type of carbon tube, and the energy increased monotonically with the increase in tensile strength. The energy curves were fitted with cubic polynomials. For different kinds of SWCNTs, when the tensile strength increased from 0 to 0.1, the energy increased to about 0.22 eV.

**Figure 12:**
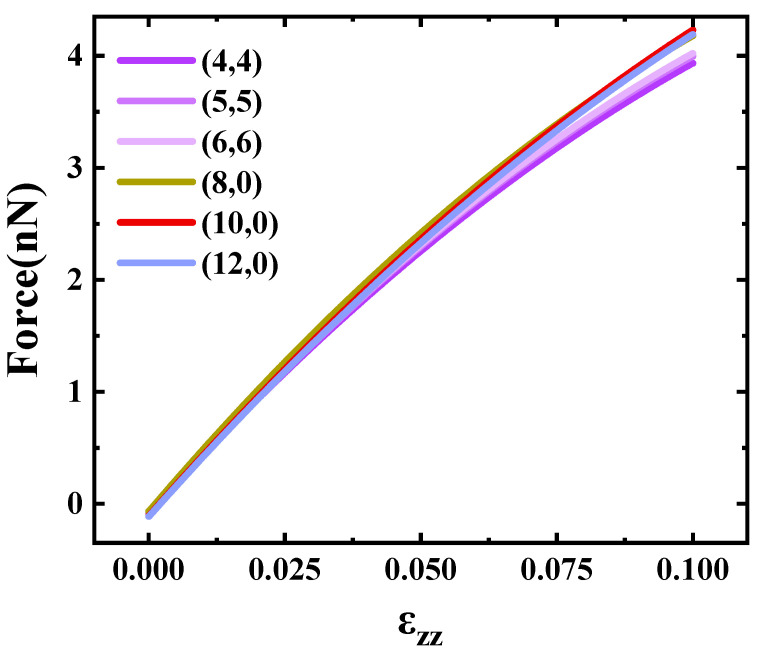
Forces required to tension SWCNTs.

The force acting on the atom can be derived as the first-order derivative of the tension energy curves. The relationship between deformation strength and atomic force is presented in Figure 12. Observing Figure 12, it is evident that the force requirement increased with the augmentation of deformation strength; the force required for tension remained nearly independent of the carbon tube type, and we found that the carbon atoms moved very little in the xy plane. When the tensile strength was 0.1, the force on the carbon atom was about 4 nN, and the force change was almost the same for all types of carbon tubes. 

### 3.3. Torsional Case

**Figure 13:**
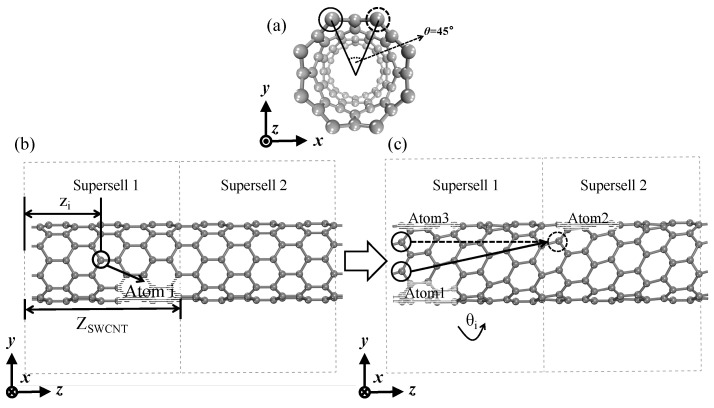
(8,0) SWCNT structure schematics in the cases of torsion (0.61 rad/nm). (**a**) Cross-section of (8,0) SWCNT; (**b**) Side view of (8,0) SWCNT before torsion; (**c**) Side view of (8,0) SWCNT after torsion.

**Figure 14:**
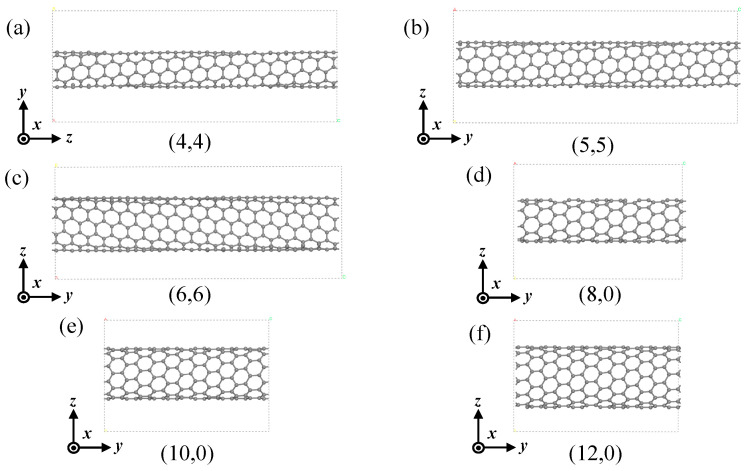
Structure diagram of six kinds of SWCNTs at minimum torsional strength. (**a**) (4,4) SWCNT (0.36 rad/nm); (**b**) (5,5) SWCNT (0.28 rad/nm); (**c**) (6,6) SWCNT (0.24 rad/nm); (**d**) (8,0) SWCNT (0.31 rad/nm); (**e**) (10,0) SWCNT (0.25 rad/nm); (**f**) (12,0) SWCNT (0.20 rad/nm).

We used a torsional strength $\alpha =\frac{\theta }{Z_{SWCNT}}$ (rad/nm) to quantify the torsional strength of SWCNTs, where *θ* is the angle of twist, as shown in Figure 13a; for an (8,0) SWCNT, *θ* = 45°, and for an (n,m) SWCNT, *θ* = 360°/n. Z_SWCNT_ represents the length of the supercell along the Z-axis, as shown in Figure 13b. Atom i underwent a central rotation of $\theta_{i}=\alpha \times z_{i}$, resulting in the torsion model on the right (Figure 13b), where z_i_ denotes the coordinate of atom i in the Z-axis direction. As shown in Figure 13c, atom 1 in supercell 1 was twisted to reach the position of atom 2 in supercell 2, which was equivalent to atom 3; the reason for this was to ensure that the model was infinitely cyclic and periodic in the Z-axis direction. Figure 14 shows six models of single-walled carbon nanotubes at the minimum torsional strength. To obtain the calculation results of a smaller torsional strength, more atoms need to be added. Due to the limited computational resources, the largest model that was able to be calculated in this paper is shown in the figure.

**Figure 15:**
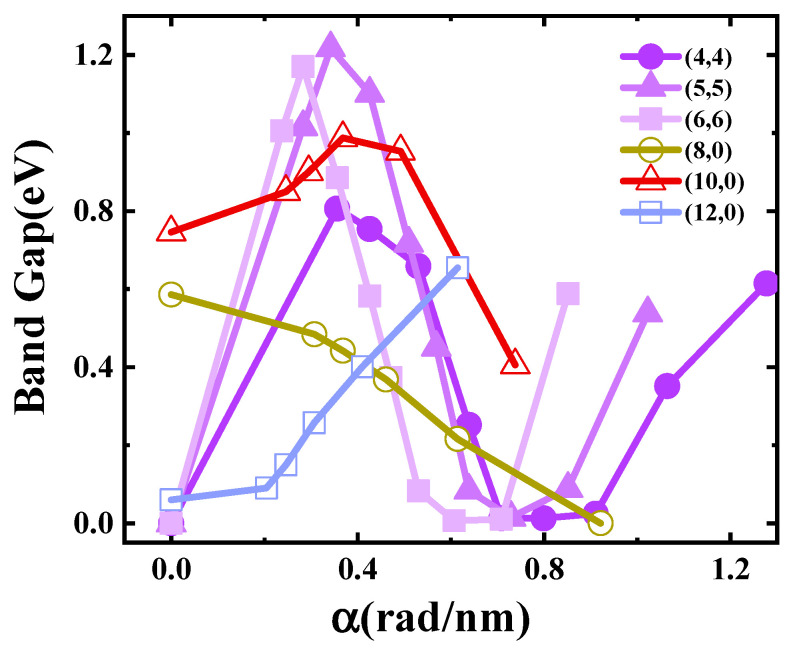
Band gaps of the SWCNTs as a function of torsional strength.

Figure 15 shows the effect of torsion on the SWCNTs’ band gap [40]. The band gaps of armchair tubes showed quite large fluctuations (metal→semiconductor→metal) with a rough period 0.8 rad/nm when the torsional strength increased from 0 rad/nm to 1.2 rad/nm. Semiconducting zigzag nanotubes (8,0) and (10,0) tended to have an overall decreasing trend in this region, while the (10,0) tube showed a small peak; when the strength reached about 0.4 rad/nm, the tubes changed from semiconductor-type carbon tubes to metal-type carbon tubes. Metallic zigzag (12,0) had an overall increasing trend, wherein the tube changed from a metal-type carbon tube to a semiconductor-type carbon tube.

**Figure 16:**
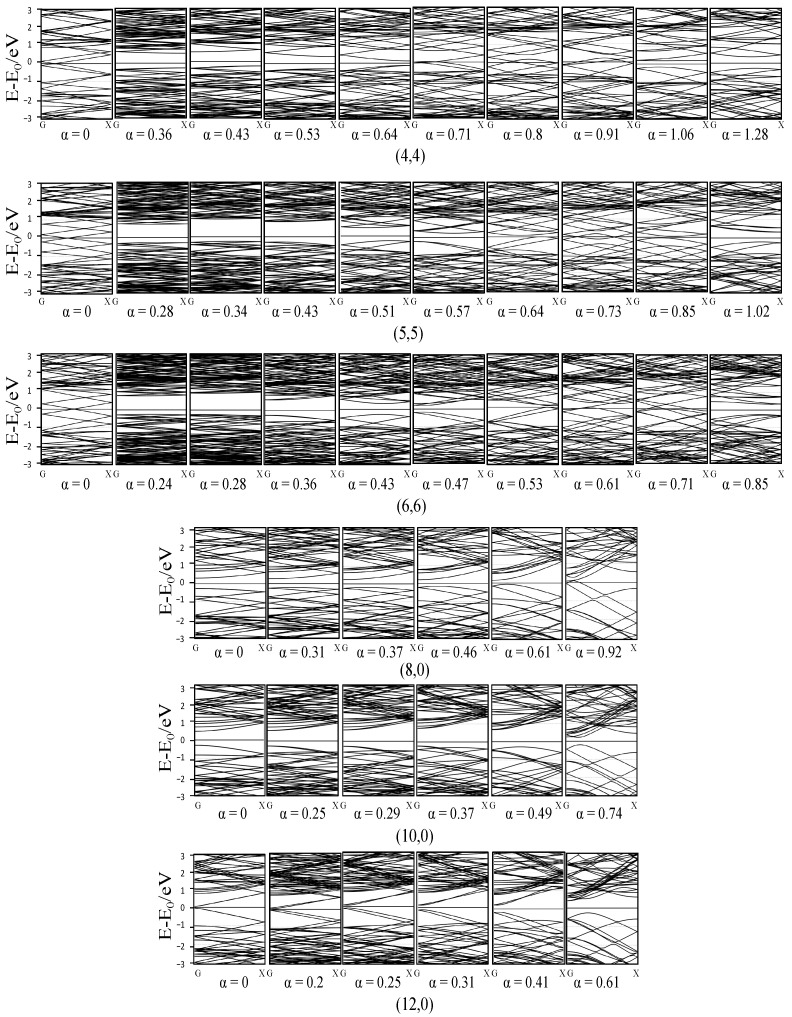
Band structure of the SWCNTs after torsion.

Figure 16 shows the effect of torsion on the SWCNTs’ band structure. We can see that different from flattening and tension, torsion did not change the band structure in a monotonic way, as the band structure will change significantly when the angle is very large. However, the monotonic trend can still be seen when the tube was slightly torqued, which can be summarized as the following: when the tube was torqued slightly, the gap tended to open for armchair tubes and metallic zigzag tubes and close for semiconducting zigzag ones.

**Figure 17:**
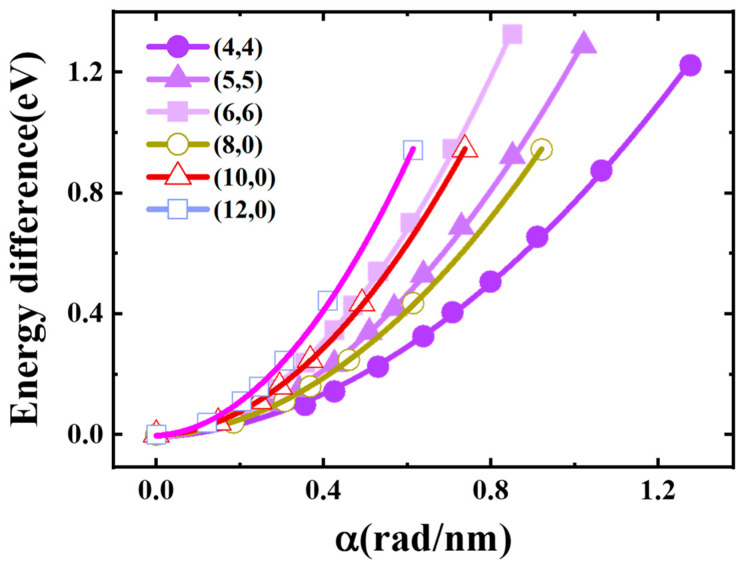
Deformation energy of the SWCNTs as a function of torsonal strength.

Figure 17 displays the fitting diagrams for the energy change of carbon atoms post-deformation. The torsion energy curves were fitted with quadratic polynomials. The deformation energy increased monotonically with the increase in torsional strength. For the same kind of carbon tube, the larger the diameter of single-walled carbon nanotubes, the more energy is required, because the energy change of a carbon tube is mainly due to the change in the distance between carbon atoms. When the same torsional strength was applied to the carbon tube, the larger the diameter of the carbon tube, the greater the relative displacement of the carbon atoms.

**Figure 18:**
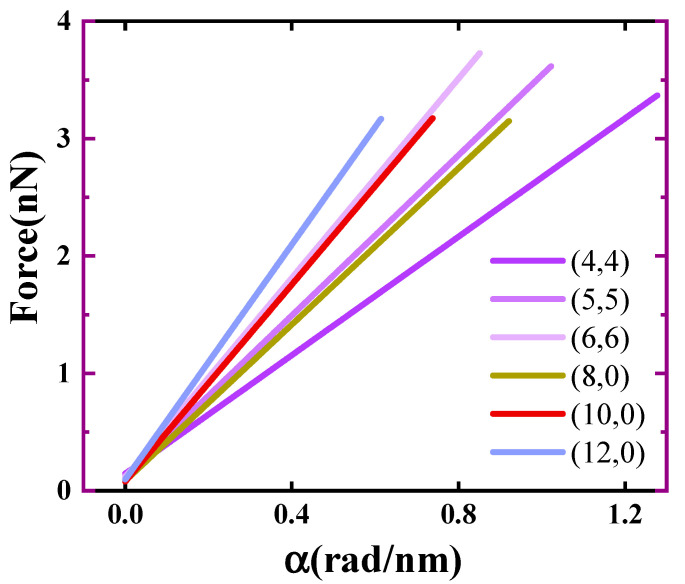
Forces required to torsion SWCNTs.

The torque was obtained from the first-order derivative of the torsion curve. The force on the carbon atom was determined by dividing the torque by the radius of the carbon tube. The relationship between deformation strength and atomic force is shown in Figure 18. Observing Figure 18, it is evident that the force requirement increased with the augmentation of deformation strength. The forces necessary for twisting SWCNTs are depicted, revealing that the twisting force increased with the diameter, indicating a direct relationship between diameter size and twisting force. For (4,4), when the torsional strength was 1.2 rad/nm, the force exerted on the carbon atom was about 3 nN, and for (12,0), when the torsional strength was 0.6 rad/nm, the force exerted on the carbon atom was about 3 nN.

### 3.4. CNTBs’ Discussion

**Figure 19:**
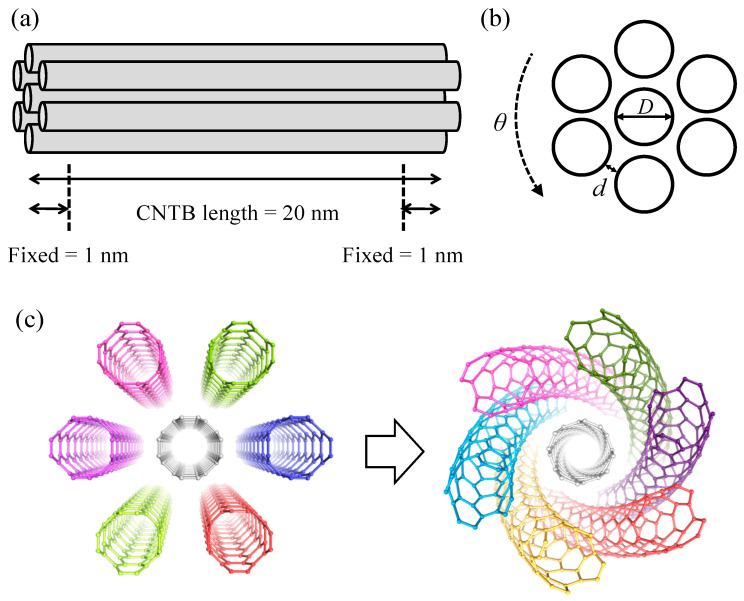
Schematics of CNTBs. (**a**) CNTBs schematic diagram before torsion; (**b**) CNTBs cross-section diagram; (**c**) CNTBs schematic diagram after torsion.

Then, we tried to estimate the band gap changes in CNTBs through mathematical models. In Figure 19a,b, the structural representation of CNTBs is shown, where seven SWCNTs were aligned together in a closed-packed shape. *θ* is the angle of twist. Figure 19c shows a diagram of the CNTBs after torsion. It should be emphasized that all seven carbon tubes were twisted. As a prototype, we chose the bundle 20 nm long with both ends fixed, shown in Figure 19. This construction method is referred to by Pereira et al. [14].

**Figure 20:**
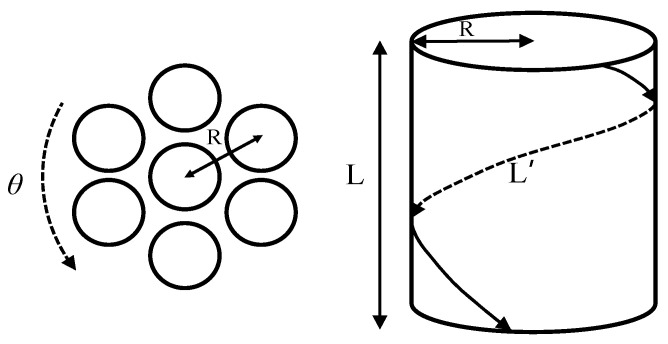
Diagram of the numerical estimation of tensile strength.

According to the dynamic simulation conducted [14], we think that the structural changes of SWCNTs in CNTBs after torsion at a small angle (0–0.6 (rad/nm)) can be represented by Figure 20. After torsioning the CNTBs, the length of the outer SWCNTs became the length of the helix of a cylinder with radius R=D+d, where “D” is the diameter of the tube and “d” is the separation distance. The tensile strength of the outer SWCNTs is defined as

$$\varepsilon_{\mathrm{zz}}=\frac{L^{\prime }-L}{L},$$

$$L^{\prime }=\sqrt{(L\times \alpha \times R{)}^{2}+{\left(L\right)}^{2}}.$$

**Figure 21:**
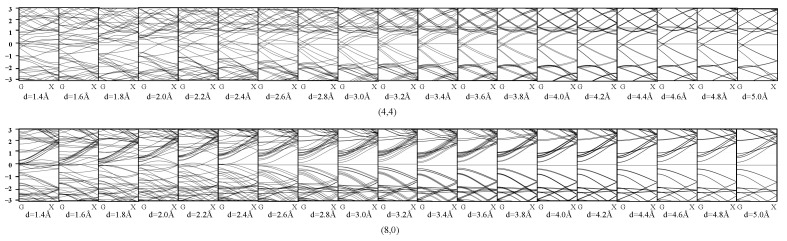
Band structure of two SWCNTS at “d” distance.

Packing of SWCNTs may lead to band-gap changes due to the interactions between tubes. If the SWCNTs are packed with a large external pressure, the adjacent tubes are in close proximity [41]. As shown in Figure 21, to analyze the effect of “d” on the band gap of CNTBs, we calculated the band gaps of two SWCNTs at distance “d”, where the “d” of (4,4) and (8,0) increased from 1.4 Å to 5 Å. The bundle structure in this paper was an isolate bunch of tubes torqued along the longitudinal axis, as illustrated in Figure 19 and Figure 20, which is somewhat different from the model by Okada et al. [42], where the bundle was crystalline. For an isolate bundle, our calculation showed that strong interaction pushed the outer tubes to the vacuum, and the corresponding distortion was alleviated. When the “d” was larger than 3.8 Å, the maximum force on each atom was less than 0.03 eV/Å, which is ignorable. In Figure 21, we can see that small inter-tube distance led to strong band hybridization, but when the value was greater than 3.8 Å, the interaction became weak and the effect was not obvious, especially regarding to the gap value. Therefore, for an isolate bunch of SWCNTs, from a qualitative point of view, we can set the inter-tube distance to 4 Å, where the distance did not change the band gap of (4,4), but for (8,0), the distance reduced the band gap by 0.0458 eV.

According to the results by Pereira et al. [14], bundles made from thin tubes did not have significant flattening deformation within a reasonable torsion. In this paper, under such a small torsional strength, the flattening effect can be neglected as well.

In such a bundle, the effective deformations of the outer tube include tension and torsion (the outer tube is bended as well, while the effect of slight bending is not considered), and inner tube is only torqued. It should be noted that the torsional strength of the outer tube is the same as that of the inner tube. We chose two tube bundles, (4,4) and (8,0), as the examples to represent metallic and semiconducting samples. Since tension tends to squeeze the gap of tube, the gap of the outer tube determines the gap of the bundle. Under small distortion, the effects of different deformations can be combined together according to the parametric model from the Huckel calculation [31].

**Figure 22:**
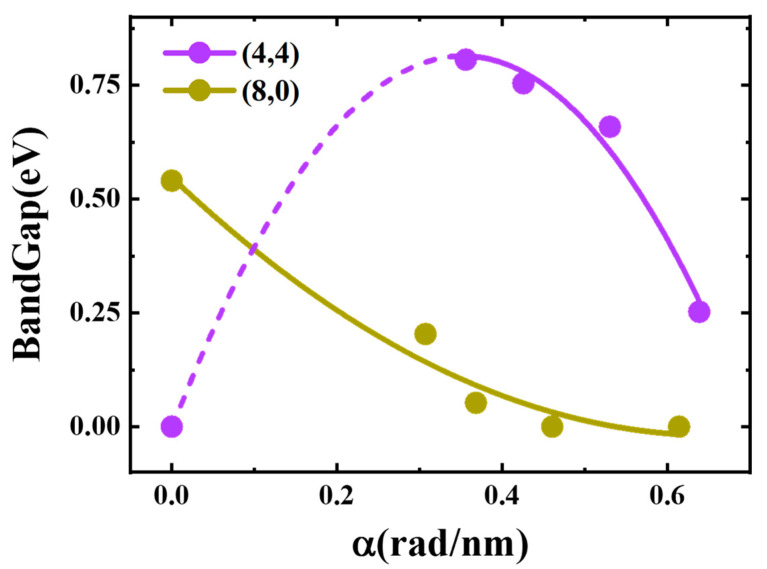
Band gaps of the CNTBs as a function of torsional strength.

When the torsional strength of a bundle was in the range of [0, 0.6], the effects of three kinds of deformation on the band gap were as follows: flattening had no effect on the band gap; tension had no effect on the band gap of (4,4), while tension decreased the band gap of (8,0); torsion firstly increased the band gap of (4,4) and then reduced the band gap, and torsion monotonically decreased the band gap of (8,0). Distance had no effect on the band gap of (4,4), but for (8,0), the distance reduced the band gap by 0.0458 eV. The final results of the bundle gap are shown in Figure 22. In the torsional calculations, due to the limit of our structural configuration, the system size needed to be very large and beyond our computational ability to simulate a small torsion parameter. Therefore, the fitting can only be done with such a sparse sampling in the region [0, 0.3]. As the final result, we can see the semiconducting (8,0) bundle had a linear declination, while the metallic (4,4) bundle showed an approximate parabola, meaning that for both metallic or semiconducting SWCNTs, it is very possible to make a metal–semiconductor or semiconductor–metal transition by bundling them together at a certain distance and applying a certain degree of torsion.

## 4. Conclusions

This study undertook a comprehensive examination of the impact of three distinct deformation mechanisms on the band gap of carbon tubes: flattening, tension, and torsion. The band-gap changes under deformations for various SWCNTs were investigated by the first-principle method. The energy change of the carbon atom was analyzed, and the force change of the carbon atom was further obtained. Combining them together, the gap change of CNTBs (20 nm length) was estimated, and transitions between metal and semiconductor were able to be foreseen during the process of bundle formation.

## Figures and Tables

**Table 1:** The k-point sampling of SWCNTs.

SWCNTs’ k-Point	Flattening	Tension	Torsion
(4,4)	(1,1,9)	(1,1,8)	(1,1,2–5)
(5,5)	(1,1,9)	(1,1,8)	(1,1,2–5)
(6,6)	(1,1,9)	(1,1,8)	(1,1,2–5)
(8,0)	(1,1,8)	(1,1,7)	(1,1,3–8)
(10,0)	(1,1,8)	(1,1,7)	(1,1,3–8)
(12,0)	(1,1,8)	(1,1,7)	(1,1,3–8)

## Data Availability

Data are contained within the article.
